# Hospitalization and medical cost of patients with elevated serum N-terminal pro-brain natriuretic peptide levels

**DOI:** 10.1371/journal.pone.0190979

**Published:** 2018-01-05

**Authors:** Toshiro Kitagawa, Noboru Oda, Mariko Mizukawa, Takayuki Hidaka, Makiko Naka, Susumu Nakayama, Yasuki Kihara

**Affiliations:** 1 Department of Cardiovascular Medicine, Hiroshima University Graduate School of Biomedical & Health Sciences, Hiroshima, Japan; 2 Heart Failure Center, Hiroshima University Hospital, Hiroshima, Japan; 3 Department of Cardiology, Hiroshima City Asa Hospital, Hiroshima, Japan; Osaka University Graduate School of Medicine, JAPAN

## Abstract

**Background:**

Patients with heart failure (HF) are reportedly at high risk for ‘all-cause’ re-hospitalization. A biomarker for HF, N-terminal pro-brain natriuretic peptide (NT-proBNP), enables to simply detect patients with possible HF (pHF). We examined the hospitalization and medical cost of Japanese patients detected by an elevated serum NT-proBNP, and also evaluated the effects of institutional team approaches for HF on their all-cause hospitalizations.

**Methods:**

We retrospectively extracted all adult patients with serum NT-proBNP ≥400 pg/ml measured between January and March 2012 in Hiroshima University Hospital as pHF-positive patients. We studied their all-cause hospitalization records during the past 3-year period. We also extracted all pHF-negative patients with NT-proBNP <400 pg/ml and studied as well. In the pHF-positive patients followed for 3 years after starting interprofessional team approaches to prevent the onset and exacerbation of HF in the hospital, we compared the hospitalization and medical cost between the 3-year periods before and after the start of the team approaches.

**Results:**

We enrolled 432 pHF-positive and 485 pHF-negative patients with one or more hospitalization records. Compared to the pHF-negative patients, the pHF-positive patients had longer total hospitalization days (median [interquartile range], 30 [13–58] versus. 18 [8–39], p <0.0001) and higher total medical cost for hospitalizations (2.42 [1.07–5.08] versus. 1.80 [0.79–3.65] million yen, p <0.0001). A subset of 303 pHF-positive patients was followed for 3 years after starting the team approaches, and we found that both total hospitalization days (30 [13–57] to 8 [0–31]) and medical cost for hospitalizations (2.59 [1.37–5.05] to 0.76 [0–2.38] million yen) showed marked reduction in them.

**Conclusions:**

Patients with an elevated serum NT-proBNP have longer hospitalizations and higher costs for all-cause hospitalizations than those without. Institutional team approaches for HF may reduce them.

## Introduction

Considerable medical resources are consumed on the care of HF (heart failure) due to repeated admissions of the patients. In a report from the United States, patients hospitalized for HF are at high risk for ‘all-cause’ re-hospitalization [1], which is considered to reflect severe comorbidities accompanied with HF [2]. The total cost of HF care in the United States exceeds $40 billion annually, with over half of these costs spent on hospitalizations [3]. In Japan, this is an emerging problem in face of drastic increases in elderly population, too. However, the real number as well as hospitalization frequency and healthcare costs of HF patients is poorly documented in Japan.

Diagnosis of HF requires a comprehensive and complicated evaluation based on subjective symptoms, objective responses, and various test findings. Even for cardiologists, it is sometimes difficult to make a clear decision about the presence and severity of HF. On the other hand, serum biomarkers have potentials to bring a simple method for detecting patients with ‘possible’ HF. Brain natriuretic peptide (BNP) and N-terminal pro-brain natriuretic peptide (NT-proBNP) are the most-established biomarkers for HF. They are secreted under conditions of pressure and volume overload in the ventricular myocardium. In the guideline for the management of HF [2], both BNP and NT-proBNP are recommended for clinical decision making regarding the diagnosis, exclusion, prognosis, and disease severity of HF. While the levels of serum BNP and NT-proBNP are affected by aging, sex, adiposity, and renal function, clinical HF is generally considered at the cut-off values of 100 pg/mL for BNP and 400 pg/ml for NT-proBNP [4]. Therefore, we may detect the majority of patients who suffer from HF by using these serum cut-off values.

In this study, we examined the conditions of medical care in Japanese patients detected by an elevated serum NT-proBNP by comparing the frequency, length, and medical costs of all-cause hospitalizations between patients with an elevated serum NT-proBNP and those without. The Heart Failure Center in Hiroshima University Hospital, founded in 2012, have focused on maintaining the HF patients’ activities of daily life and preventing the readmission through the interprofessional team approaches. Thus, we evaluated the effects of the institutional team approaches for HF on their all-cause hospitalizations.

## Methods

### Study population

We conducted the retrospective study with patients receiving medical care at Hiroshima University Hospital in Japan. This institution has 32 medical departments and 740 hospital beds, and have introduced the Japanese bundled payment system for hospitalized care costs (DPC/PDPS; Diagnosis Procedure Combination / Per-Diem Payment System). Under this system, basic hospital stays, tests, diagnostic imaging, medication and injections, and treatments under 1000 points (currently 10000 yen) are reimbursed with inclusive payments set for each diagnosis-related group (prospective payment on a per-diem basis), while medical care, surgery, anesthesia, radiation therapy and treatments over 1000 points area reimbursed on the basis of the fee-for-service system [5].

According to the flow chart for the diagnosis of HF with natriuretic peptides shown in the guideline from the European Society of Cardiology (the latest version at the beginning of this study) [4], we defined the serum NT-proBNP ≥400 pg/ml as a possible HF (pHF) condition. We extracted all adults (age ≥20 years old) with the serum NT-proBNP ≥400 pg/ml measured at the central chemical laboratory in Hiroshima University Hospital between January and March 2012 (ECLIA; electrochemiluminescent immunoassay, Roche Diagnostic) as pHF-positive patients. In patients with one or more measurements of serum NT-proBNP in the 3-month period, the maximum value was used for the extraction. We studied their all-cause hospitalization records during the past 3-year period (from January 2009 to December 2011), including the primary diagnosis, admitted department, hospitalization days, and medical cost for each hospitalization. In addition, we extracted all adults with NT-proBNP <400 pg/ml measured during the same period as pHF-negative patients and studied as well. The pHF-negative patients were considered as controls. For all the enrolled patients, we collected additional information including body mass index (BMI), hemoglobin level (Hb), and estimated glomerular filtration rate (eGFR), which influenced the level of serum NT-proBNP [6, 7]. As a basic parameter in clinical practice, the cardiothoracic ratio (CTR) on chest x ray was also recorded. In patients with echocardiogram findings acquired between January and March 2012, the left ventricular ejection fraction (LVEF) was recorded.

The central chemical laboratory in Hiroshima University Hospital has adopted the NT-proBNP, not BNP, as the measurement item of serum brain natriuretic peptide since February 2009. Accordingly, there were no measurements of serum BNP between January and March 2012.

### Studied indexes for hospitalization

In this study, we selected patients with one or more hospitalization records in Hiroshima University Hospital during the past 3-year period. In each patient, we filed a total number of hospitalizations during the 3-year period and length of stay for one hospitalization. We defined the ‘Repeated-Hospitalization’ as ≥4 hospitalizations during the 3-year period (numerically equal to >1 hospitalizations/year). Additionally, we calculated the total hospitalization days during the 3-year period in each patient. As for the healthcare costs, we filed medical cost for one hospitalization and total medical cost for all the hospitalizations during the 3-year period (yen) in each patient.

### Effect of team approaches on hospitalizations

The Heart Failure Center in Hiroshima University Hospital was founded in January 2012. This organization is composed of multidisciplinary medical care personnel of the hospital, including cardiologists, cardiovascular surgeons, nurses, physical therapists, nutritionists, pharmacists, and social workers. It has provided actual care to patients with clinical HF, and also promoted efforts to prevent the occurrence and recurrence of symptomatic HF through various approaches. S1 Table shows the specific approaches provided by the Heart Failure Center. In addition, to support the patient self-managing, all members have performed lifestyle guidance using our established notebook for HF management. All members routinely have taken a meeting for a case conference, and sought to collaborate on their approaches. Notably, subjects receiving these approaches includes patients without clinical HF (Stage A or B HF). In Japan, the team medical care itself for HF management generates no additional medical cost, but some approaches (rehabilitation, nutritional guidance, and drug administration guidance) need cost individually.

In the pHF-positive patients who were extracted between January and March 2012 and were followed at Hiroshima University Hospital between April 2012 and March 2015, we additionally collected the information regarding all-cause hospitalizations in the 3-year period after starting team approaches for HF prevention and management by the Heart Failure Center. A total number of hospitalizations, total hospitalization days, and total medical cost for all the hospitalizations were compared between the 3-year periods before and after the start of the team approaches. For this comparison, we included the patients who died during the study period, because they mostly needed medical resources for hospitalizations in the period, and those were presumably considerable from the perspective of medical economy.

### Statistical analysis

The serum NT-pro BNP level and continuous variables regarding hospitalizations are expressed as median value and interquartile range as they show non-parametric distributions, and other continuous values are expressed as mean ± standard deviation. Student *t* test or Mann-Whitney *U* test was used to compare groups in terms of continuous variables. Wilcoxon signed-rank test was used to compare the paired continuous variables between the 3-year periods before and after the start of team approaches for HF by Heart Failure Center. Categorical variables are reported as number (proportion, %) and were compared using Pearson’s chi-square test. Pearson correlation coefficient was used to calculate the correlation between hospitalization days and medical cost for hospitalization. Linear regression was used to examine the association of the clinical factors including elevated serum NT-proBNP (≥400 pg/ml) with hospitalization days and medical cost for multivariate analysis adjusted for age, sex, BMI, Hb, eGFR, CTR, the presence or absence of the primary diagnosis related to malignancy, and LVEF (only for patients with echocardiogram data). All analyses were done using JMP 10.0.1 statistical software (SAS Institute Inc, North Carolina). A p value of <0.05 was considered statistically significant.

### Ethical concerns

Prior to the current study, Hiroshima University institutional review board approved this retrospective study. Because the study protocol was based on the extraction and review of medical records with the anonymous fashion, the institutional review board waived the need for patient consent.

## Results

### Baseline information

We studied 432 pHF-positive and 485 pHF-negative patients with one or more hospitalization histories during the past 3-year period. The serum NT-proBNP levels in the pHF-positive and pHF-negative patients were 1125 (690–2999) and 114 (58–241) pg/ml, respectively.

Table 1 shows the baseline information of the studied patients and hospitalizations. The pHF-positive patients were older than the pHF-negative patients, and sex ratio was similar between the groups. Body mass index was lower and CTR was higher in the pHF-positive patients. In the subgroup with echocardiogram findings, LVEF was lower in the pHF-positive patients. Hemoglobin level did not differ between the groups, but eGFR was lower in the pHF-positive patients. Total number of hospitalization records was 2103. This included 1149 (54.6%) admissions to other than the cardiovascular medicine, cardiovascular surgery, and emergency departments. In both groups, more than half of the hospitalizations were to other than the cardiovascular medicine, cardiovascular surgery, and emergency departments (53.8% and 55.5%, respectively). The pHF-positive patients had more HF as the primary diagnosis (12.8%), but the pHF-negative patients also had 3.9% HF as the primary diagnosis. As for the disease area of the primary diagnosis, more than 40 percent were related to cardiology and more than half were related to non-cardiovascular diseases in both groups. The pHF-positive patients had more renal diseases as the primary diagnosis, while the pHF-negative had more hematologic diseases. The pHF-positive patients had less primary diagnoses related to malignancy.

**Table 1:** Baseline information of studied patients and hospitalizations.

	pHF-positive	pHF-negative	p values
Number	432	485	
Age (years)	70 ± 12	66 ± 13	< 0.0001
Male sex	269 (62.3)	301 (62.1)	0.95
BMI (kg/m^2^)	22.9 ± 4.1	23.5 ± 3.8	0.046
Hemoglobin level (g/dL)	12.3 ± 2.5	12.5 ± 2.2	0.12
eGFR (ml/min/1.73m^2^)	55.9 ± 43.0	68.8 ± 29.0	< 0.0001
CTR (%)	54.0 ± 7.0	48.8 ± 6.2	< 0.0001
LVEF (%)	53.4 ± 14.0 (n = 380)	60.7 ± 9.0 (n = 409)	< 0.0001
Total number of hospitalizations	1054	1049	
Hospitalization in cardiovascular medicine, cardiovascular surgery, and emergency departments	487 (46.2)	467 (44.5)	0.44
HF as the primary diagnosis	135 (12.8)	41 (3.9)	< 0.0001
Disease area of the primary diagnosis			
Cardiology	457 (43.4)	461 (44.0)	0.79
Hematology	82 (7.8)	156 (14.9)	< 0.0001
Gastroenterology	119 (11.3)	108 (10.3)	0.46
Pneumology	97 (9.2)	84 (8.0)	0.33
Neurology	46 (4.4)	53 (5.1)	0.46
Nephrology	64 (6.1)	7 (0.7)	< 0.0001
Rheumatology and Orthopedics	28 (2.7)	26 (2.5)	0.80
Endocrinology	18 (1.7)	15 (1.4)	0.61
Primary diagnosis related to malignancy	247 (23.4)	321 (30.6)	0.0002

Categorical data are presented as number (%), and continuous data are presented as mean ± standard deviation.

BMI, body mass index; CTR, cardiothoracic ratio; eGFR, glomerular filtration rate; HF, heart failure; pHF, possible heart failure.

### Hospitalization frequency, length, and medical cost

**Fig 1:**
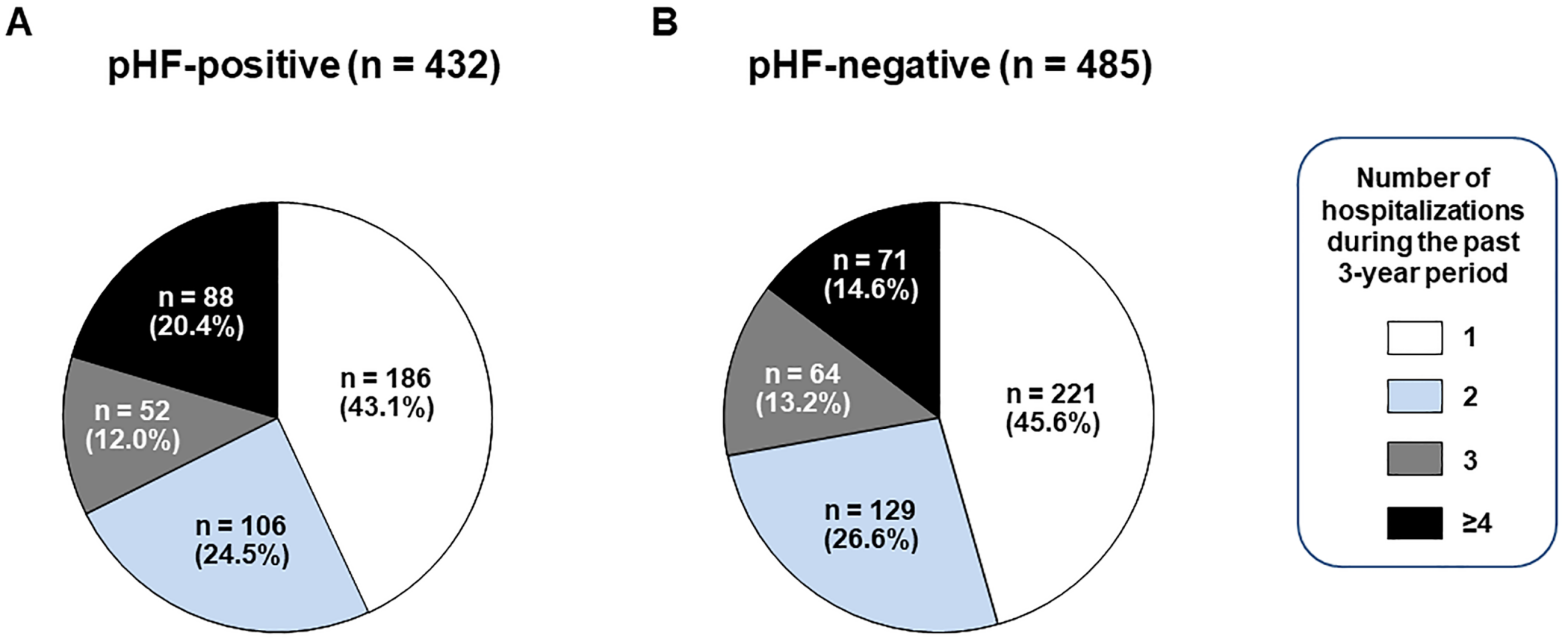
Distribution of the number of all-cause hospitalizations. The possible heart failure (pHF)-positive patients (A) had higher rate of the ‘Repeated-Hospitalization’ (≥4 times during the 3-year period) than the pHF-negative patients (B) (20.4 vs. 14.6%, p = 0.02).

Table 2 shows the hospitalization frequency, length, and medical costs during the past 3-year period of the pHF-positive and negative patients. Fig 1 shows the distribution of hospitalization frequency in the two groups. The frequency of hospitalization was similar between the groups, while the pHF-positive patients showed significantly higher rate of the ‘Repeated-Hospitalization’ than the pHF-negative patients (20.4 vs. 14.6%, p = 0.02). The pHF-positive patients had longer length per one hospitalization in all departments or in the cardiovascular medicine, cardiovascular surgery, and emergency departments. Consequently, the pHF-positive patients had longer total hospitalization days during the past 3-year period than the pHF-negative patients. As for the medical cost for hospitalizations, the pHF-positive patients needed higher cost per one hospitalization in all departments or in the cardiovascular medicine, cardiovascular surgery, and emergency departments. Consequently, the pHF-positive patients needed higher total cost for hospitalizations during the past 3-year period than the pHF-negative patients.

**Table 2:** Hospitalization data of the past 3-year period.

Variables	pHF-positive (n = 432)	pHF-negative (n = 485)	p values
Frequency of hospitalization (times)	2 (1–3)	2 (1–3)	0.12
Repeated-Hospitalization	88 (20.4)	71 (14.6)	0.02
Length for one hospitalization in all departments (days)	13 (6–24)	8 (3–18)	< 0.0001
Length for one hospitalization in cardiovascular medicine, cardiovascular surgery, and emergency departments (days)	12 (4–26)	4 (2–12)	< 0.0001
Total hospitalization days (days during 3 years)	30 (13–58)	18 (8–39)	< 0.0001
Medical cost for one hospitalization (million yen)	0.84 (0.45–1.81)	0.72 (0.30–1.54)	< 0.0001
Medical cost for one hospitalization in cardiovascular medicine, cardiovascular surgery, and emergency departments (million yen)	1.17 (0.49–2.22)	1.00 (0.26–1.86)	< 0.0001
Total medical cost for hospitalizations (million yen)	2.42 (1.07–5.08)	1.80 (0.79–3.65)	< 0.0001

Categorical data are presented as number (%), and continuous data are presented as mean ± standard deviation.

BMI, body mass index; CTR, cardiothoracic ratio; eGFR, glomerular filtration rate; HF, heart failure; pHF, possible heart failure.

On multivariate analysis adjusted for age, sex, BMI, Hb, eGFR, CTR, and the presence or absence of the primary diagnosis related to malignancy, the high NT-proBNP value (≥400 pg/ml) remained as a significant factor for increasing total hospitalization days (β = 0.13, p = 0.0003) and total medical cost for hospitalizations (β = 0.12, p = 0.0018) (Table 3).

**Table 3:** Linear regression analysis to examine the association of the clinical factors with hospitalization days and medical cost.

**With hospitalization days**				
Variables	Univariate		Multivariate	
	β	p values	β	p values
Age (years)	-0.02	0.54		
Male sex	-0.04	0.18		
BMI (kg/m^2^)	-0.07	0.027	-0.03	0.32
Hemoglobin level (g/dL)	-0.25	< 0.0001	-0.26	< 0.0001
eGFR (ml/min/1.73m^2^)	-0.002	0.94		
CTR (%)	0.06	0.059	0.03	0.39
Primary diagnosis related to malignancy	0.01	0.74		
High NT-proBNP value (≥400 pg/ml)	0.16	< 0.0001	0.13	0.0003
**With medical cost for hospitalizations**				
Variables	Univariate		Multivariate	
	β	p values	β	p values
Age (years)	-0.03	0.32		
Male sex	0.03	0.36		
BMI (kg/m^2^)	-0.04	0.17		
Hemoglobin level (g/dL)	-0.15	< 0.0001	-0.18	< 0.0001
eGFR (ml/min/1.73m^2^)	-0.01	0.69		
CTR (%)	0.08	0.013	0.07	0.053
Primary diagnosis related to malignancy	0.02	0.63		
High NT-proBNP value (≥400 pg/ml)	0.15	< 0.0001	0.12	0.0018

BMI, body mass index; CTR, cardiothoracic ratio; eGFR, glomerular filtration rate; NT-proBNP, N-terminal pro-brain natriuretic peptide.

In the subgroup in which LVEF was available (n = 789), univariate analysis revealed that LVEF did not correlate with increased total hospitalization days (β = -0.03, p = 0.37) and total medical cost for hospitalizations (β = -0.06, p = 0.080). After additional adjustment for LVEF, the high NT-proBNP value (≥400 pg/ml) remained as a significant factor for increasing total hospitalization days (β = 0.15, p = 0.0002) and total medical cost for hospitalizations (β = 0.14, p = 0.0005).

### Relationship between hospitalization length and medical cost

**Fig 2:**
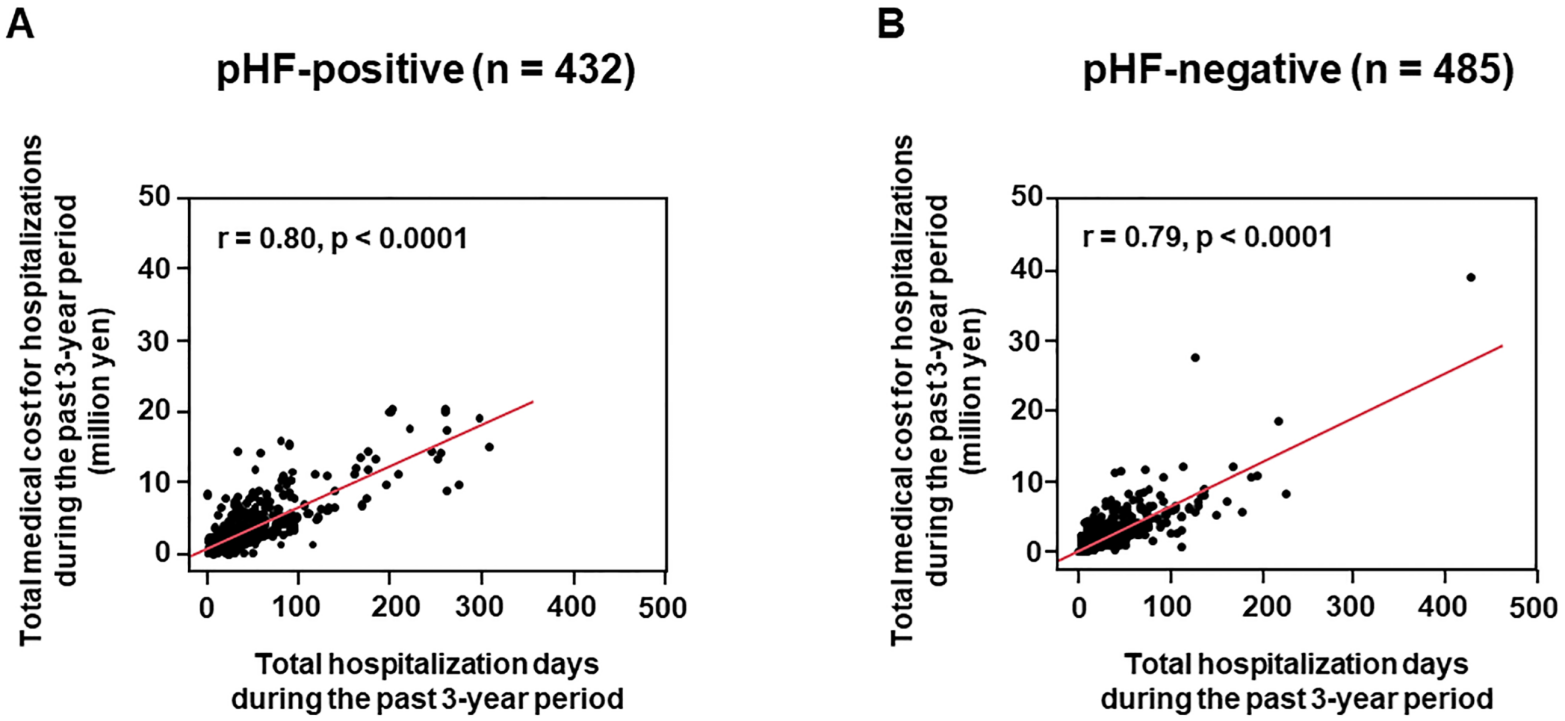
Correlation of total medical cost for all-cause hospitalizations with total hospitalization days during the past 3-year period. In both the possible heart failure (pHF)-positive (A) and negative patients (B), there was a positive strong correlation between total medical cost and hospitalization days.

Fig 2 shows the correlation between total hospitalization days and medical cost during the past 3-year period in the pHF-positive and negative patients. The total medical cost showed a positive strong correlation with the total hospitalization days, in both groups. The correlated level was similar between the groups (r = 0.80, p <0.0001 and r = 0.79, p <0.0001, respectively).

### Hospitalizations after the start of team approaches

Of the 432 pHF-positive patients, 401 were followed at Hiroshima University Hospital for 3 years after starting team approaches for HF prevention and management by the Heart Failure Center. We further selected 303 patients who were managed in the department of cardiovascular medicine or cardiovascular surgery, because they received the interprofessional team approaches for HF (rehabilitation, education, and lifestyle guidance, etc.) individually as needed in the 3 years. Their 3-year mortality rate was 8.3% (25/303). During the follow-up period, 105 patients (34.7%) had no hospitalization. The total number of hospitalization records was 463. This included 239 (51.6%) admissions to other than the cardiovascular medicine, cardiovascular surgery, and emergency departments.

In these pHF-positive patients, we compared all-cause hospitalization records between the 3-year periods before and after starting the team approaches by the Heart Failure Center (Table 4). The frequency of hospitalization, total hospitalization days, and total medical cost for all the hospitalizations were markedly reduced in the latter period. Additionally, when we further selected the patients with serum NT-proBNP ≥1000 pg/ml (n = 168) or ≥2000 pg/ml (n = 101), we found that both subgroups also showed marked reduction in those parameters of hospitalizations.

**Table 4:** Hospitalizations of pHF-positive patients before and after the start of team approaches for HF patients.

NT-proBNP level	Variables	Before (January 2009–December 2011)	After (April 2012–March 2015)	p values
≥400 pg/ml (n = 303)	Frequency of hospitalization (times)	2 (1–3)	1 (0–2)	< 0.0001
	Total hospitalization days	30 (13–57)	8 (0–31)	< 0.0001
	Total medical cost for hospitalizations (million yen)	2.59 (1.37–4.98)	0.76 (0–2.38)	< 0.0001
≥1000 pg/ml (n = 168)	Frequency of hospitalization (times)	2 (1–3)	1 (0–2)	< 0.0001
	Total hospitalization days	31 (14–74)	10 (0–41)	< 0.0001
	Total medical cost for hospitalizations (million yen)	2.53 (1.31–5.05)	1.03 (0–2.62)	< 0.0001
≥2000 pg/ml (n = 101)	Frequency of hospitalization (times)	2 (1–4)	1 (0–2)	< 0.0001
	Total hospitalization days	48 (25–81)	15 (0–49)	< 0.0001
	Total medical cost for hospitalizations (million yen)	3.22 (1.73–7.03)	1.54 (0–3.18)	< 0.0001

Continuous data are presented as median value (interquartile range).

NT-proBNP, N-terminal pro-brain natriuretic peptide; pHF, possible heart failure.

## Discussion

We studied the conditions of medical care, especially hospitalization, for patients detected by an elevated serum HF biomarker, NT-proBNP. Although a single-center study, our results demonstrate the frequent and longer medical care and higher costs for all-cause hospitalizations of Japanese patients with higher NT-proBNP than those without. More than half of the hospitalizations were to non-cardiovascular departments, indicating that such patients were likely to be hospitalized due to both cardiovascular and non-cardiovascular problems. We found a positive correlation between their hospitalization days and medical costs for hospitalizations under the Japan’s own unique system for hospitalized care costs. This suggests that a main factor increasing medical resources needed on their medical care is the frequency and length of hospitalization. After starting the interprofessional team approaches for HF prevention and management in the hospital, the frequency and length of hospitalization and costs for hospitalizations showed marked reduction. The introduction of institutional team approaches for HF may reduce their all-cause hospitalizations and medical costs.

This study used a simple one-point measurement of serum biomarker for extracting the pHF-positive patients. However, there is no single diagnostic test for HF, and one-point serum level of NT-proBNP cannot clearly discriminate HF patients from non-HF patients. Thus, the pHF-positive cohort, defined by higher NT-proBNP, presumably included substantial patients who did not qualify for the category of clinical syndrome of HF, while the pHF-negative cohort included patients in whom clinical HF was suspected but excluded, and those with stable chronic HF. Nevertheless, we found major differences in hospitalization days and medical cost during a 3-year period, even when based on classification only by the level of serum NT-proBNP. The measurement of serum NT-proBNP has a potential to identify the patients with cardiac overload who should be under a careful management to prevent hospitalization and under an intensive care to reduce hospitalization days. In addition, serum NT-proBNP is stable and exhibits little variance in measurement between examiners and institutions. Thus, the use of serum NT-proBNP should have several advantages in clinical care.

To assess medical resources for patients with an elevated serum NT-proBNP, we investigated the frequency, length, and medical cost for all-cause hospitalizations. In the patients, more than half of the hospitalizations were primarily related to non-cardiovascular diagnosis, indicating such patients frequently had medical problems other than cardiovascular diseases. The fact that the proportion of hospitalizations due to ‘cardiology’ diagnosis is similar between the pHF-positive and negative cohorts seems counterintuitive, but our result is consistent with the report from the United States showing that more than half of the hospitalizations in HF patients are related to non-cardiovascular causes [8]. We demonstrate that the patients with higher serum NT-proBNP have longer hospitalization days and higher medical costs for all-cause hospitalizations during the past 3-year period than those without. From the perspective of the relationship between serum NT-proBNP level and renal function, the lower eGFR and more renal diseases in the pHF-positive cohort seem plausible (Table 1). However, the linear regression analysis showed that eGFR was not a significant factor for increasing hospitalization length and costs (Table 3). On the one hand, after adjustment for the conditions of anemia and renal function, the presence of elevated serum NT-proBNP independently increases both hospitalization length and costs. This suggests that the patients with an elevated serum NT-proBNP need more medical resources than those without, irrespective of the conditions of anemia and renal function. A recent report also suggests the clinical value of NT-proBNP measurement independently of the level of renal function [9]. Furthermore, the results from the subgroup analysis in which LVEF is available indicate that an elevated serum NT-proBNP correlates with more medical resources irrespective of left ventricular ejection fraction.

Several epidemiological studies have been conducted in Europe and the United States to reveal the real conditions of medical resources for patients with chronic HF, which are documented in the recent guideline [2]. As for studies about Japanese HF patients, the Japanese Cardiac Registry of Heart Failure in Cardiology (JCARE-CARD) enrolled 2,675 patients with chronic HF receiving inpatient treatment at 164 institutions nationwide, and assessed patient background, severity, treatment, and outcomes [10]. The Heart Institute of Japan-Department of Cardiology (HIJC) is a registry-based follow-up study of patients with HF admitted to 15 institutions in 8 prefectures, suggesting the necessity of further improvements in the treatment of Japanese HF patients [11]. The Chronic Heart Failure Analysis and Registry in the Tohoku District (CHART) study analyzed 1,154 patients with stable HF in the Tohoku District, and sought to reveal the prognosis and predictors for mortality of chronic HF patients in Japan [12]. These are large-scale studies focusing on Japanese HF patients, but does not clarify how much medical resources have been used. While our study deals with patients detected only by an elevated serum HF biomarker, it may reflect one aspect of higher medical resources needed on patients suffering from HF.

Japan has a unique payment system for medical cost (combination of prospective per-diem basis and fee-for-service system). Under this Japanese condition, this study showed that the medical costs for hospitalizations increase according to the hospitalization length in both pHF-positive and negative patients. The correlation of the hospitalization costs to length was similar between the two groups. The ‘long stay in hospitals’ itself was a fundamental factor to increase medical cost for the pHF-positive patients. To reduce the medical resources for them, we should set two targets; one is to prevent repeated admissions, and the other is to shorten the hospitalization length. The approach to these targets should contribute to the improvement in cost-effectiveness of Japanese healthcare system.

The Heart Failure Center in Hiroshima University has been appreciated as a specific interprofessional team for the management of HF patients. Actual medical care to patients by the interprofessional members is its main work, while the promotion of efforts in the whole hospital to prevent new-onset HF and exacerbation of HF is its important mission. Based on the problem and requirement for each patient, it has provided the rehabilitation for aerobic exercise, patient education about HF, and lifestyle guidance for reducing the risks of HF occurrence and recurrence by the interprofessional members (S1 Table). In this study, we found that hospitalization frequency, length, and costs of the pHF-positive patients were markedly reduced after the start of such activities by the Heart Failure Center. This result highlights a beneficial effect of the introduction of institutional team approaches on reduction in medical resources required for their all-cause hospitalizations. Such approaches could be helpful for general disease management mainly through lifestyle modification. On the one hand, the subgroups with much higher NT-proBNP (≥1000 pg/ml or ≥2000 pg/ml) are considered to include more patients with cardiac overload and clinical HF, and the effect in them can be also expected according to our results. A systemic review of randomized trials suggests that multidisciplinary strategies for the management of HF patients are expected to reduce their mortality and all-cause hospitalizations [13]. In face of the ‘heart failure pandemic’ [14], how to manage patients with chronic HF has been a worldwide issue from a clinical and social perspective, and the ‘team approach’ could be a key strategy for improvement in the management of those patients.

This study has limitations. First, it is hard for one-point measurement of the biomarker to reflect the clinical course of each patient. Temporal high level of serum NT-proBNP may be incidentally influenced by the acute extracardiac factors, such as acute inflammatory diseases. However, this study, although it is retrospective, suggests that this one-point blood test may screen the population which needs high consumption of medical resources. Second, this is a retrospectively-designed study and does not clarify the predictive value of measuring serum NT-proBNP to detect the HF patients with higher medical resources. Third, we had no specific information about the team approaches for each patient. Thus, the detailed reasons for the reduction in medical resources of patients with an elevated serum NT-proBNP are unknown from the current study, and a prospective cohort of patients receiving direct interprofessional team approaches is needed to investigate them. As in the previous report regarding HF patients [15], whether nurse-led HF management can reduce the number of cardiac events, readmissions, and hospitalization length in patients detected by an elevated serum NT-proBNP is worth investigating in the future. Focusing on their social background of patients, such as life style, family support, dietary & drug management, and exercise habit, might be helpful for development of an effective multidisciplinary strategy for them [16]. Finally, this is a single-center study, and cannot reflects the wide-area condition. We believe the current study provides important messages about the real condition and management of patients with pHF, but a large-scale cohort applying the serum NT-proBNP measurement is required in the future.

In conclusion, the patients with an elevated serum NT-proBNP have frequent and longer hospitalizations and more medical costs for all-cause hospitalizations as compared with those without. Their medical costs increase as the longer hospitalization days. While the fact that the pHF-positive cohort does not correspond to patients with clinical HF should be cautioned, the current study indicates the longer medical care and higher costs for hospitalizations of Japanese patients with an elevated serum HF biomarker. Medical resources required for their all-cause hospitalizations could be markedly reduced through institutional team approaches, even though the main focuses are HF prevention and management.

## Supporting information

S1 Table Specific approaches by the heart failure center. (DOC)

## References

[1] Krumholz HM, Merrill AR, Schone EM, Schreiner GC, Chen J, Bradley EH, et al Patterns of hospital performance in acute myocardial infarction and heart failure 30-day mortality and readmission. Circ Cardiovasc Qual Outcomes. 2009;2:407–413. doi: 10.1161/CIRCOUTCOMES.109.883256 20031870 10.1161/CIRCOUTCOMES.109.883256

[2] Yancy CW, Jessup M, Bozkurt B, Butler J, Casey DE Jr, Drazner MH, et al 2013 ACCF/AHH guideline for the management of heart failure: A report of the American College of Cardiology Foundation/American Heart Association Task Force on practice guidelines. Circulation. 2013;128:e240–327. doi: 10.1161/CIR.0b013e31829e8776 23741058 10.1161/CIR.0b013e31829e8776

[3] Go AS, Mozaffarian D, Roger VL, Benjamin EJ, Berry JD, Borden WB, et al Heart disease and stroke statistics—2013 update: a report from the American Heart Association. Circulation. 2013;127:e6–e245. doi: 10.1161/CIR.0b013e31828124ad 23239837 10.1161/CIR.0b013e31828124ad PMC5408511

[4] Dickstein K, Cohen-Solal A, Filippatos G, McMurray JJ, Ponikowski P, Poole-Wilson PA, et al ESC guidelines for the diagnosis and treatment of acute and chronic heart failure 2008: the Task Force for the diagnosis and treatment of acute and chronic heart failure 2008 of the European Society of Cardiology. Developed in collaboration with the Heart Failure Association of the ESC (HFA) and endorsed by the European Society of Intensive Care Medicine (ESICM). Eur Heart J. 2008;29:2388–2442. doi: 10.1093/eurheartj/ehn309 18799522 10.1093/eurheartj/ehn309

[5] Ishii M. DRG/PPS and DPC/PDPS as prospective payment systems. Japan Med Assoc J. 2012;55:279–291. unspecified. 25237234

[6] Palazzuoli A, Masson S, Ronco C, Maisel A. Clinical relevance of biomarkers in heart failure and cardiorenal syndrome: the role of natriuretic peptides and troponin. Heart Fail Rev. 2014;19:267–284. doi: 10.1007/s10741-013-9391-x 23563622 10.1007/s10741-013-9391-x

[7] Fukuta H, Ohte N, Mukai S, Saeki T, Kobayashi K, Kimura G. Anemia is an independent predictor for elevated levels of natriuretic peptides in patients undergoing cardiac catheterization for coronary artery disease. Circ J. 2008;72:212–217. doi: 10.1253/circj.72.212 18219156 10.1253/circj.72.212

[8] Wang G, Zhang Z, Ayala C, Wall HK, Fang J. Costs of heart failure-related hospitalizations in patients aged 18 to 64 years. Am J Manag Care. 2010;16: 769–776. unspecified. 20964473

[9] Scrutinio D, Mastropasqua F, Guida P, Ammirati E, Ricci V, Raimondo R, et al Renal dysfunction and accuracy of N-terminal pro-B-type natriuretic peptide in predicting mortality for hospitalized patients with heart failure. Circ J. 2014;78:2439–2446. 25168191 10.1253/circj.cj-14-0405

[10] Tsutsui H, Tsuchihashi-Makaya M, Kinugawa S, Goto D, Takeshita A, JCARE-CARD Investigators. Clinical characteristics and outcome of hospitalized patients with heart failure in Japan. Circ J. 2006;70:1617–1623. doi: 10.1253/circj.CJ-14-0405 17127810 10.1253/circj.70.1617

[11] Kawashiro N, Kasanuki H, Ogawa H, Matsuda N, Hagiwara N. Clinical characteristics and outcome of hospitalized patients with congestive heart failure: results of the HIJC-HF registry. Circ J. 2008;72:2015–2020. doi: 10.1253/circj.CJ-08-0323 18931450 10.1253/circj.cj-08-0323

[12] Shiba N, Watanabe J, Shinozaki T, Koseki Y, Sakuma M, Kagaya Y, et al Analysis of chronic heart failure registry in the Tohoku district: third year follow-up. Circ J. 2004;68:427–434. doi: 10.1253/circj.68.427 15118283 10.1253/circj.68.427

[13] McAlister FA, Stewart S, Ferrua S, McMurray JJ. Multidisciplinary strategies for the management of heart failure patients at high risk for admission: A systematic review of randomized trials. J Am Coll Cardiol. 2004;44:810–819. doi: 10.1016/j.jacc.2004.05.055 15312864 10.1016/j.jacc.2004.05.055

[14] Starling RC. The heart failure pandemic: changing patterns, costs, and treatment strategies. Cleve Clin J Med. 1998;65:351–358. unspecified. 9679390 10.3949/ccjm.65.7.351

[15] Strömberg A, Mårtensson J, Fridlund B, Levin LA, Karlsson JE, Dahlström U. Nurse-led heart failure clinics improve survival and self-care behaviour in patients with heart failure: results from a prospective, randomised trial. Eur Heart J. 2003;24:1014–1023. doi: 10.1016/S0195-668X(03)00112-X 12788301 10.1016/s0195-668x(03)00112-x

[16] Takabayashi K, Ikuta A, Okazaki Y, Ogami M, Iwatsu K, Matsumura K, et al Clinical characteristics and social frailty of super-elderly patients with heart failure—The Kitakawachi Clinical Background and Outcome of Heart Failure Registry. Circ J. 2016;81:69–76. doi: 10.1253/circj.CJ-16-0914 27904019 10.1253/circj.CJ-16-0914

