# Supplementary material for: Hospitalization and medical cost of patients with elevated serum N-terminal pro-brain natriuretic peptide levels
Source: PLoS One. 2018 Jan 5;13(1):e0190979. doi: 10.1371/journal.pone.0190979 (PMC5755927; doi:10.1371/journal.pone.0190979)
Supplement: S1 Table — (DOC) [file pone.0190979.s001.doc]

**S1 Table. Specific Approaches by the Heart Failure Center.**

| Approaches | Personnel responsible | Content |
| --- | --- | --- |
| Patient education | Physicians and nurses | Individual interview and lecture class about HF |
| Telenursing | Nurses | Physiological telemonitoring and teleconsultation |
| Inpatient/outpatient rehabilitation | Physical therapists | Providing aerobic exercise program |
| Nutritional guidance | Nutritionists | Guidance focusing mainly on salt reduction |
| Drug administration guidance | Pharmacists | Teaching and advising for correct medication |
| Consultation on daily life | Medical social workers | Supporting the use of public nursing-care services |
